# Synthesis, Anticancer Screening of Some Novel Trimethoxy Quinazolines and VEGFR2, EGFR Tyrosine Kinase Inhibitors Assay; Molecular Docking Studies

**DOI:** 10.3390/molecules26102992

**Published:** 2021-05-18

**Authors:** Abdulmalik S. Altamimi, Adel S. El-Azab, Sami G. Abdelhamid, Mubarak A. Alamri, Ashraf H. Bayoumi, Safar M. Alqahtani, Alhumaidi B. Alabbas, Ali I. Altharawi, Manal A. Alossaimi, Menshawy A. Mohamed

**Affiliations:** 1 Department of Pharmaceutical Chemistry, College of Pharmacy, Prince Sattam bin Abdulaziz University, Alkharj 11942, Saudi Arabia; mubalamri@gmail.com (M.A.A.); safar.alqatani@psau.edu.sa (S.M.A.); ab.alabbas@psau.edu.sa (A.B.A.); a.altharawi@psau.edu.sa (A.I.A.); m.alossaimi@psau.edu.sa (M.A.A.); 2 Department of Pharmaceutical Chemistry, College of Pharmacy, King Saud University, Riyadh 11451, Saudi Arabia; adelazaba@hotmail.com; 3 Department of Pharmaceutical Chemistry, Faculty of Pharmacy, Al-Azhar University, Cairo 11884, Egypt; s.gaber51@gmail.com; 4 Department of Pharmaceutical Organic Chemistry, Faculty of Pharmacy, Al-Azhar University, Cairo 11884, Egypt; ashraf.bayoumi@azhar.edu.eg

**Keywords:** synthesis, methoxy quinazoline, anticancer, docetaxel, VEGFR2, EGFR

## Abstract

A new series of 8-methoxy-2-trimethoxyphenyl-3-substituted quinazoline-4(3)-one compounds were designed, synthesized, and screened for antitumor activity against three cell lines, namely, Hela, A549, and MDA compared to docetaxel as reference drug. The molecular docking was performed using Autodock Vina program and 20 ns molecular dynamics (MD) simulation was performed using GROMACS 2018.1 software. Compound 6 was the most potent antitumor of the new synthesized compounds and was evaluated as a VEGFR2 and EGFR inhibitor with (IC50, 98.1 and 106 nM respectively) compared to docetaxel (IC50, 89.3 and 56.1 nM respectively). Compounds 2, 6, 10, and 8 showed strong cytotoxic activities against the Hela cell line with IC50 of, 2.13, 2.8, 3.98, and 4.94 µM, respectively, relative to docetaxel (IC50, 9.65 µM). Compound 11 showed strong cytotoxic activity against A549 cell line (IC50, 4.03 µM) relative to docetaxel (IC50, 10.8 µM). Whereas compounds 6 and 9 showed strong cytotoxic activity against MDA cell line (IC50, 0.79, 3.42 µM, respectively) as compared to docetaxel (IC50, 3.98 µM).

## 1. Introduction

Cancer is one of the world’s most prominent health-related diseases, which makes the search for safe and efficient anticancer compounds a major goal for researchers. Many heterocyclic compounds have been intensively studied to classify new anticancer agents. Quinazoline is one of the most common groups of heterocyclic compounds used in medicine because of its broad variety of biological activities. We are particularly interested in the present work with quinazoline derivatives that have been identified as a class of heterocyclic cancer chemotherapeutic agents with significant therapeutic effects [1,2]. For example, the effects of 2-chloro-4-anilino quinazoline derivatives as dual inhibitors for VEPGR and EGFR [3]. Some methoxy quinazolines are active as anticancer compounds [4]. Quinazoline-isatin conjugates as an epidermal growth factor inhibitor [5]. 2-substituted mercapto-3-(3,4,5-trimethoxybenzyl)-4(3*H*)-quinazolinone analogues show antitumor activity [6]. Trimethoxyanilide based on 4 (3*H*)-quinazolinone scaffolds as an antitumor compound [7]. Some quinazolinone nuclei are frequently used in medicinal chemistry [8,9,10,11,12,13,14,15,16,17,18,19,20,21,22,23,24]. Some substituted 2-mercapto-4(*3H*)-quinazolinones and 2,3-disubstitued 4(*3H*)-quinazolinone derivatives work as anti-inflammatory and analgesic agents, such as COX-1/2 inhibitory activities [9,10]. Two identical anilinoquinazoline rings with ethylaminoethyl linker and *N*-ethylaminomorpholine moiety to enhance water solubility were synthesized and evaluated as inhibitors of epidermal growth factor receptor (EGFR) tyrosine kinase with IC (50) values in the low micromolar range [25]. Some dimers and substituted amino derivatives of quinazoline were synthesized, docked into ATP binding sites of epidermal growth factor receptor (EGFR) tyrosine kinase, and evaluated as antitumor compounds against MCF7 cells with IC50 = 0.06 μg/mL; 1.64 nmol/mL [26]. Benzofuran and indole derivatives of 4-aminoquinazoline are inhibitors of epidermal growth factor receptor (EGFR) tyrosine kinase phosphorylation. A heterocyclic scaffold has been found to lead to heterocyclic quinazolines with good biological activity against a panel of EGFR-positive cell lines, such as the human lung cancer (A549), epithelial colorectal adenocarcinoma (Caco-2), hepatocellular carcinoma (C3A), breast adenocarcinoma (MCF-7), and cervical cancer (Hela) cell lines with reduced side effects [27].

**Figure 1:**
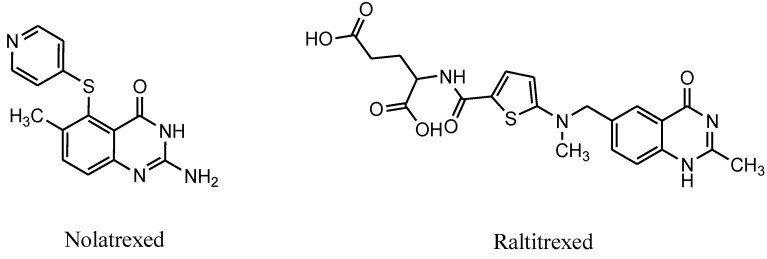
Chemical structure for some quinazoline derivatives used as anticancer drugs, such as nolatrexed and Raltitrexed.

Quinazoline has become an important pharmacophore for anticancer research since the recent FDA approval of some quinazoline derivatives as anticancer drugs, such as Raltitrexed (TomudexTM) and nolatrexed (thymitaqTM) as shown in Figure 1, as well as gefitinib (IressaTM), lapatinib (TykerbTM also known as GW-572016), Vandetanib (ZactimaTM), afatinib (GilotrifTM), and erlotinib (TarcevaTM). 

**Figure 2:**
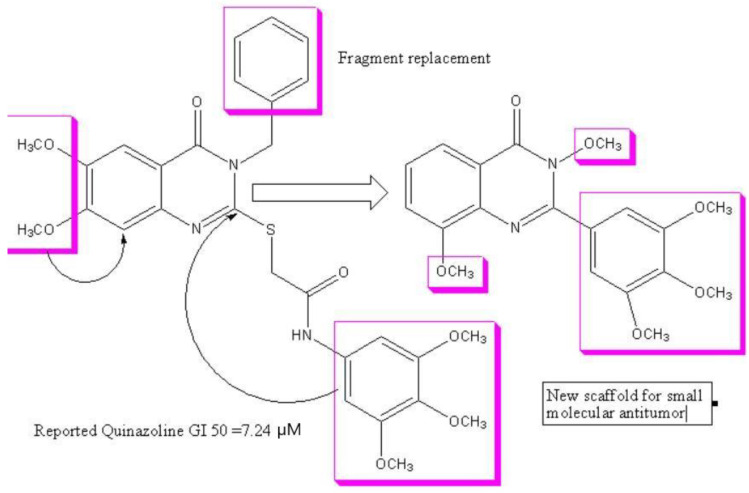
Reported quinazoline with GI_50_ µg/mL [15] and designed quinazoline derivatives as antitumor compounds.

In our present study, we designed a number of quinazoline derivatives bearing trimethoxy phenyl moiety in position 2 and insertion of methoxy at position 8 to give different electronic, steric, and lipophilic characters that might help improve the antitumor activity of the newly designed scaffolds. In addition, we kept the substitution at position 3 in an attempt to get optimized quinazoline derivatives with promising anticancer activity (Figure 2).

## 2. Results and Discussion

### 2.1. Chemistry

**Scheme 1:**
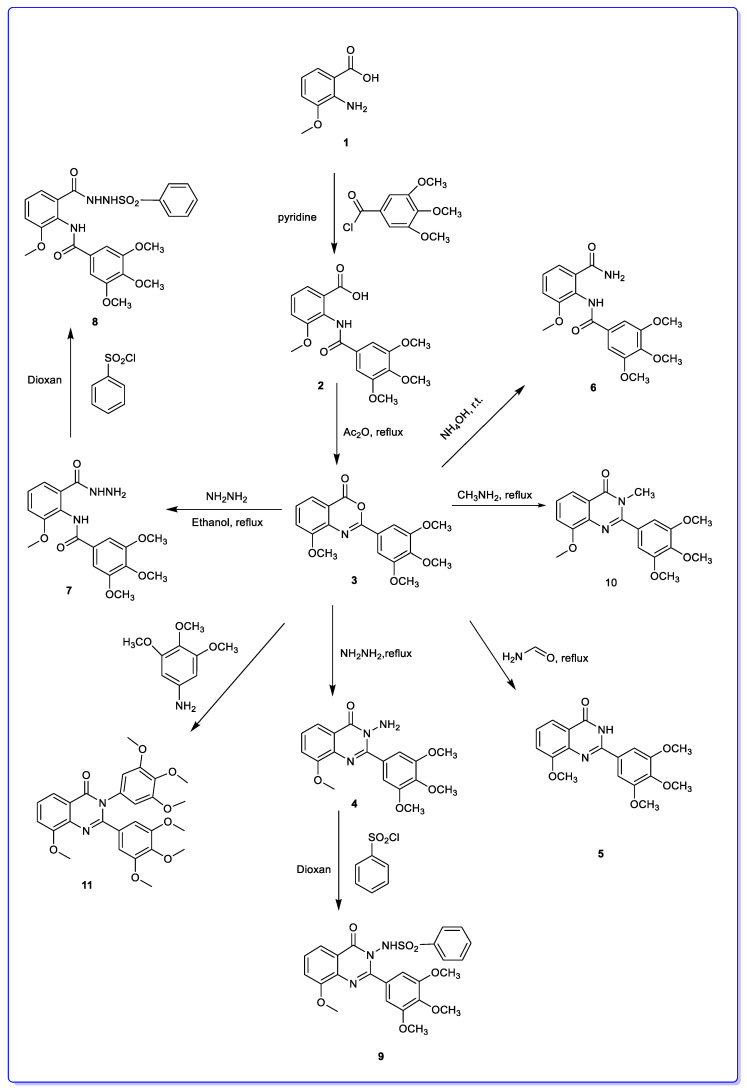
Synthesis of new quinazoline derivatives 2–11.

The synthesis of the target compounds is depicted in scheme 1. The starting material 8-methoy-2-trimethoxyphenyl-4*H*-benzo[d](1,3)oxazin-4-one (**3**) was prepared according to a reported procedure [28] by allowing *N*-(trimethoxybenzoyl)-3-methoxyanthranilic acid to boil with acetic anhydride, then compound **3** allowed was fused with hydrazine to get 3-amino-8-methoxy-2-trimethoxyphenylquanazoline-4(*3H*)-one (**4**). IR spectra of compound **4** showed absorption bands at 3221.35, 3387.83, and 1655.19 cm^−1^ due to stretching vibration of the NH_2_ group and CO, respectively, and ^1^H NMR led to the appearance of a singlet of two protons for the NH_2_ group at 5.842. Compound 3 reacted with formamide to give 8-methoxy-2-trimethoxyphenylquanazoline-4(*3H*)-one (**5**), the latter compound showed a stretching band for CO at 1655.67 cm^−1^, and ^1^H NMR showed a singlet peak at 8.292 ppm for the NH group. Compound **3** reacted with ammonia to create benzamide derivative 6, IR of compound **6** showed stretching vibration bands for NH_2_ and NH at 3193.13, 3367.91, and 3389.62 and stretching bands at 1672.66 and 1663.23 for two CO groups. Moreover the ^1^H NMR data showed a singlet peak for the NH_2_ group at 6.069 ppm and a singlet peak at 9.782 ppm for NH moiety. Compound **3** reacted with hydrazine hydrate in the presence of ethanol to get hydrazide derivative **7**. Compound **7** showed characteristic CO bands of diamide at 1670.63 and 1655.19 and NH, NH_2_ absorption at 3376.45, 3382.21, 3175.43, and 3295.34 cm^−1^, in addition to an ^1^H NMR singlet peak at 4.479 ppm for NH_2_ and two singlets for NH of diamide at 10.134 and 10.167 ppm. Compound 7 reacted with benzene sulphonyl chloride, which afforded compound **8** with absorption bands of NH groups at 3366.45, 3372.52, and 3385.43 cm^−1^ and CO band at 1670.65 and 1655.37 cm^−1^ on the IR spectrum, as well as singlets at 8.5497 and 12.615 ppm for NH protons in ^1^H NMR. Additionally, compound **4** reacted with benzene sulphonyl chloride to give compound **9** with absorption bands at 1655.82 and 3385.56 cm^−1^ due to Co and NH, respectively, beside the ^1^H NMR singlet for NH at 11.885 ppm. Moreover, compound **3** refluxed with methyl amine to give 3-methyl-8-methoxy-2-trimethoxyphenylquanazoline-4(*3H*)-one (**10**), the latter compound was confirmed by the presence of the CH_3_ group at 17.08 and 2.637 ppm in ^13^C NMR and ^1^H NMR, respectively. Compound **3** reacted with 3, 4, 5-trimethoxy aniline to create 3-(3, 4, 5-trimethoxyphenyl)-8-methoxy-2-trimethoxyphenylquanazoline-4(*3H*)-one (**11**), which was confirmed by the presence of singlet peaks at 56.18, 59.56, and 60.23 ppm and 3.624, 3.768, and 3.964 ppm due to one methoxy group and two trimethoxy groups in ^13^C NMR and ^1^H NMR spectrum, respectively (Scheme 1).

### 2.2. VEGFR2 and EGFR Inhibitory Activity

The synthesized compounds were evaluated for anticancer activity by adapting a reported procedure [29,30,31,32,33] using two enzymes, VEGFR2 and EGFR, as well as three cell lines. The inhibitory activity for VEGFR2 and EGFR was assessed for the most active one (compound **6**) and the results are reported as IC_50_ values. Compound No. **6** proved to have almost 91% inhibitory activity for enzyme VEGFR2 compared to the inhibitory activity of the reference drug, with IC_50_ values 98.1 to compound **6** and 89.3 for docetaxel for the positive control. Meanwhile, the inhibitory activity for enzyme EGFR was almost 53%, with IC_50_ value 106 compared to IC _50_ value 56.1 for docetaxel as reference drug (Table 1). 

**Figure 3:**
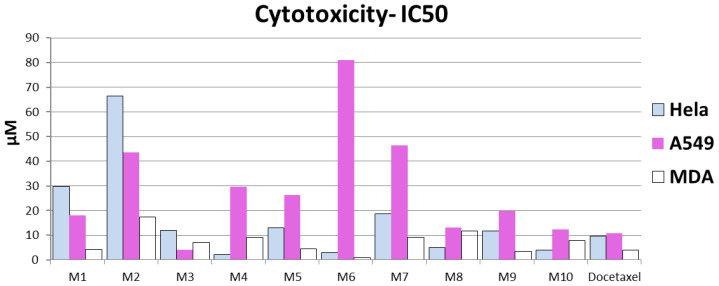
Representative graph showing the most active compounds in every cell line, where Hela cell line columns are in blue, A549 cell line columns are in purple, and MDA cell line columns are in white.

The synthesized compounds were also subjected to anticancer assay using three cell line panels. The first cell line was Hela cells using docetaxel as positive control and the results were recorded as IC_50_ values. Compounds **2**, **6**, **10**, and **8** proved to be the most active Hela cells inhibitors, with IC_50_ values indicating that they are more potent than docetaxel, with scores of IC_50_, 2.13, 2.8, 3.98, 4.94 µM, respectively, and 3-methoxy-*N*-(trimethoxyphenyl) anthranilic acid was the most potent of them as a Hela inhibitor, with almost fivefold inhibitory activity. Compounds **9**, **11**, and **5** proved to have good inhibitory activity, with IC_50_ values of 11.70, 12.00, and 12.90 µM, while compounds **4** and **3** had moderate activity as Hela cell inhibitors, with IC_50_ values 29.80 and 66.50 as compared with that of docetaxel IC_50_, 9.65 µM (Table 2 and Figure 3). The second one was A549, using docetaxel as positive control, and the results were determined as IC_50_ values. 8-methoxy-2-trimethoxymethyl-3-trimethoxyphenyl quanazoline-4(*3H*)-one (**11**) proved to be the most potent one of them, with almost two-and-a-half-fold more than the reference drug, with an IC_50_ value of 4.03 µM. Compounds **10**, **8**, **4**, and **9** showed good A549 inhibitory activity, with IC_50_ values 12.30, 13.00, 18.00, and 19.90 µM, respectively. Compounds **5**, **2**, **3**, and **7** showed moderate A549 inhibitory activity, with IC_50_ values of 26.30, 29.70, 43.60, and 46.30 µM, respectively. In addition, compound **6** proved to be the least active one, with IC_50_, 81.00 µM in comparison to docetaxel (IC_50_, 10.8 µM) (Table 2 and Figure 3).

The third cell line was MDA cells using a positive control and the results are reported as IC_50_ values. Compounds **6** and **9** proved to be the ones that were more potent than the positive control, with IC_50_ values of 0.79 and 3.42 µM, respectively, and compound **6** (3-methoxy-2-(trimethoxybenzamido) benzamide) was the most active of all as MDA inhibitor, with a fourfold higher inhibitory activity compared to the reference drug. Respectively, compounds **4** and **5** had good inhibitory activity with MDA cells with IC_50_, 4.12 and 4.39 µM. Compounds **11**, **7**, **2**, and **8** showed moderate inhibitory activity, with IC_50_ values 7.06, 9.00, 9.24, and 11.70 µM, respectively. In addition, compound **3** showed the least activity, with an IC_50_ value of 17.40 µM as compared with docetaxel IC_50_, 3.98 µM. (Table 2 and Figure 3).

### 2.3. Molecular Docking Analysis

**Figure 4:**
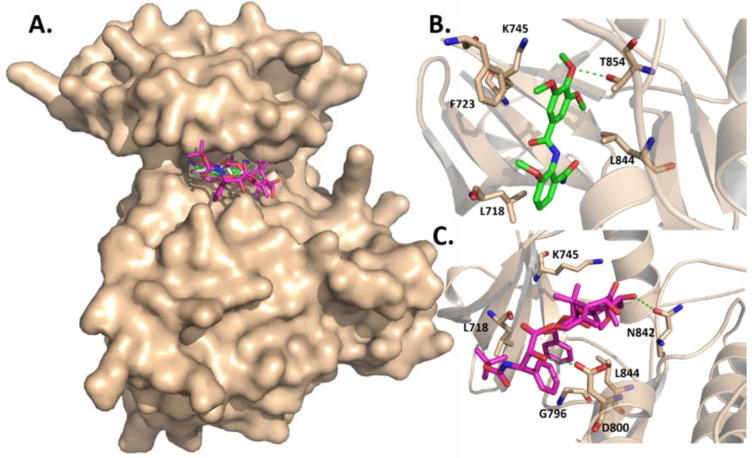
The binding of M6 to EGFR ^L858R/T790M^ tyrosine kinase (PDB ID: 6S9B). (**A**) Surface representation for the binding mode of M6 (green), docetaxel (pink), and co-crystallized ligand compound 1 (white) to EGFR tyrosine kinase. Ribbon representation for the binding interaction of (**B**) M6 (green) and (**C**) docetaxel (pink) with EGFR tyrosine kinase. Hydrogen bonds are shown in green dashed lines.

In order to get an insight into the mechanism of interaction between M6 and EGFR and VEGFR2 tyrosine kinases, a molecular docking analysis was performed using docetaxel and co-crystalized ligands as references. The docking of M6 into the ATP pocket of EGFR^L858R/T790M^ indicates that M6 adapted a binding mode similar to docetaxel as well as the co-crystal ligand; compound 1 had a docking energy score of −7.4 kcal/mol (Figure 4A). Compound 1 is a potent non-covalent inhibitor of EGFR ^del19 T790M C797S^, with an IC_50_ = 790 nM [34].

**Figure 5:**
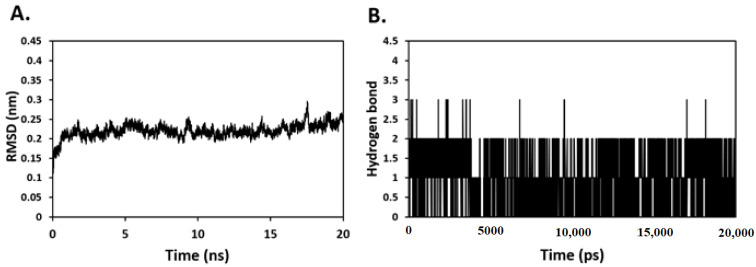
(**A**) The backbone RMSD of M6-EGFR complex for 20 ns MD simulation. (**B**) Total number of molecular hydrogen bonds for the M6-EGFR complex over the simulation time.

M6 was able to form a hydrogen bond between the oxygen atom of methoxy-group and T854 within the hinge region and hydrophobic interaction with side chains of F723, L718, and L844 (Figure 4B). Notably, a mutation of L718 within the P-loop has been reported as a mutation conferring resistance to osimertinib [35]. It was also in close proximity to catalytic lysine residue, K745. In contrast, the docetaxel was involved in two hydrogen bonds, with the nitrogen of N842 and oxygen of D800, as well as several hydrophobic interactions with L844, G796, and L718 (Figure 4C). To confirm the docking results, M6–EGFR complex was subjected to a 20 ns MD simulation to further analyze the molecular mechanism of the ligand–protein interaction. The backbone RMSD of the M6–EGFR complex was calculated to predict the M6–EGFR complex stability over time. As shown in Figure 4A, the complex reached equilibration after ~1.5 ns with a normal fluctuation pattern for the rest of the time (~18.5 ns) between 0.2 and 0.3 Å, indicating a stable dynamic behavior (Figure 5A). The hydrogen bonds are major contributors to the stability of the binding between the protein and the ligand. M6 was found to form up to three hydrogen bonds with the active site of EGFR over the simulation time (Figure 5B).

**Figure 6:**
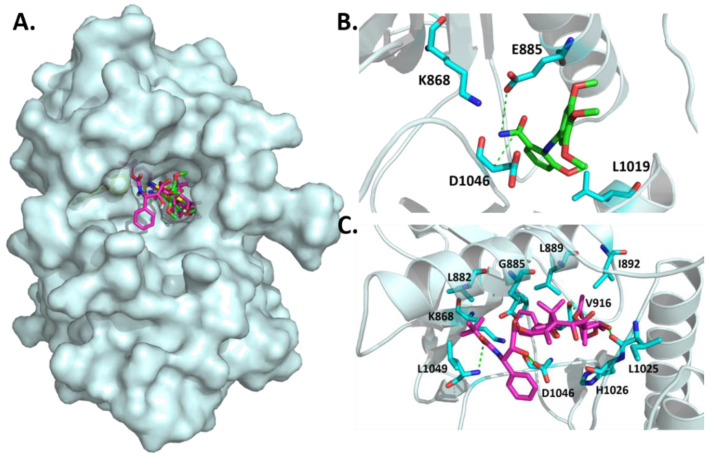
The binding of M6 to VEGFR2 tyrosine kinase (PDB ID: 4ASD). (**A**) Surface representation for the binding mode of M6 (green), docetaxel (pink), and co-crystallized sorafenib (yellow) to VEGFR2 tyrosine kinase. Ribbon representation for the binding interaction of (**B**) M6 (green) and (**C**) docetaxel (pink) with VEGFR2 tyrosine kinase. Hydrogen bonds are shown in green dashed lines.

Similarly, the docking of M6 against the wild type VEGFR2 was performed using sorafenib as a reference. Sorafenib is a nonselective potent ATP-competitive inhibitor of VEGFR2 in which IC_50_ = 2.3 nM [36]. The results indicate that M6 binds to the ATP binding pocket with a docking energy score of −7.3 kcal/mol. This compound binds to the conserved regions of the ATP site, similarly to docetaxel as well as the co-crystalized ligand, sorafenib (Figure 6A). However, unlike M6, docetaxel was able to bind the regulatory domain pocket (RDP), as was the sorafenib, as both molecules have larger substituents [37].

**Figure 7:**
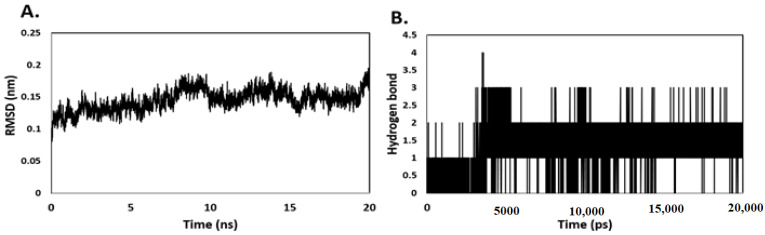
(**A**) The backbone RMSD of M6-VEGFR2 complex for 20 ns MD simulation. (**B**) Total number of molecular hydrogen bonds for the M6-VEGFR2 complex over the simulation time.

In term of interactions, M6 was involved in two hydrogen bonds between the nitrogen and oxygen atoms within the benzamide moiety and E885 of the αC helix; as well as with the D1046 residue in the DFG region of the VEGFR2, respectively; and in hydrophobic bonds with the side chain of L1019 (Figure 6B). On the other hand, docetaxel made four hydrogen bonds with V916, L1025, D1046, AND L1049, as well as hydrophobic interactions with K868, L882, G885 L889, I892, and H1026 (Figure 6C). The stability of the M6-VEGFR2 complex was confirmed by MD simulation. The backbone RMSD of the complex showed a normal dynamic behavior with a fluctuation range between 0.1 and 0.2 Å over the 20 ns simulation time. Additionally, this complex formed up to three stable hydrogen bonds over simulation period (Figure 7).

The MD summation results suggest a stable binding of M6 within the ATP binding pocket of EGFR and VEGFR2 due to the stable hydrogen bonds formed with the proteins. These results are in line with the docking and in vitro results supporting the notion that this compound might act as a dual EGFR and VEGFR2 tyrosine kinase inhibitor.

## 3. Materials and Methods

### 3.1. Chemistry

All melting points were determined in open capillaries and are uncorrected. Microanalyses were conducted on a Perkin–Elmer 2408 analyzer (Perkin Elmer, USA). Thin layer chromatography was performed on Merck 5 × 10 cm plates. Samples were percolated with silica gel GF254 using (EtOAc, hexane 1:10) as the solvent system and a short wavelength UV light for visualization. All fine chemicals and reagents used were purchased from (Aldrich chemical Co. U.S.A.) 1 HNMR samples were recorded on a Bruker 500 MHz spectrophotometer (Bruker AXS Inc., Switzerland). Chemical shifts are in δ (ppm) values downfield from tetramethyl siloxane as an internal standard. The mass spectra were measured on Waters Micromass (Water AQUITY UPLC System LCT Premier XE Serial no: KE468, USA).

3-methoxy-*N*-(trimethoxyphenyl) anthranilic acid (**2**)

A mixture of 3-methyl anthranilic acid (1, 2.37 g, 0.01 mol) and 3,4,5-trimethoxybenzoyl chloride (0.01 mol) in dry pyridine (50 mL) was heated under reflux for 12 h, then cooled and poured onto dilute HCL. The separated solid was filtered, washed with water, dried, and recrystallized from MeOH; 1H NMR (500 MHz, DMSO-*d*_6_); δ 3.732 (s, 3H, phenyl-OCH3), 3.85 (s, 9H, phenyl-OCH3), 7.22 (d, 1H, *J* = 6.0 Hz, phenyl-H), 7.43 (d, 2H, *J* = 5.5 Hz, phenyl-H), 7.506 (d, H, *J* = 8.5 Hz, quinazoline-H), 7.80–7.81 (d, 1H, *J* = 5.5 Hz, quinazoline-H), 7.82–7.84 (d, 1H, *J* = 6.5 Hz, quinazoline-H), 7.96 (s, 2H, phenyl-H), 9.79 (s, 1H, NHCO), 10.17 (s, 1H, -COOH); 13C NMR (125 MHz, DMSO-*d*_6_) d; 56.08, 60.12, 105.21, 114.86, 121.25, 125.73, 126.84, 129.48, 130.35, 139.89, 152.41, 154.74, 164.60, 167.25. MS (*m*/*z*): [M+1]: 362.1215.

8-methoxy-2-trimethoxyphenyl-4*H*-benzo[d](1,3)oxazin-4-one (**3**) 

Cyclization of *N*-(trimethoxybenzoyl)-3-methoxyanthranilic acid (2, 2.37 g, 0.01 mol) was performed by boiling in (50 mL) acetic anhydride, after which it was cooled and poured onto water. The separated solid was filtered, dried, and recrystallized from MeOH; yield, 83%; mp: 182–184 °C; IR (KBr) ν max/cm^−1^ 1655.11 (CO); 1H NMR (500 MHz, DMSO-*d*_6_); δ 3.631 (s, 3H, phenyl-OCH3), 3.73 (s, 9H, phenyl-OCH3), 7.21–7.25 (m, 3H, quinazoline-H),) 7.26 (s, 2H, phenyl-H), 9.81 (s, 1H, -CONH); 13C NMR (125 MHz, DMSO-*d*_6_) δ 56.08, 60.12, 105.21, 114.86, 121.25, 125.73, 126.84, 129.48, 130.35, 139.89, 152.41, 154.74, 164.60, 167.25. MS (*m*/*z*): [M+1]: 344.1112.

3-amino-8-methoxy-2-trimethoxyphenylquanazoline-4(*3H*)-one (**4**)

A mixture of 4*H*-1,3-benzoxazin-4-one derivative (3, 3.43 g, 0.01 mol) and hydrazine (15 mL) was fused for 6 h, poured in water, filtered, dried, and recrystallized from MeOH; yield, 81%; mp: 182–184 °C; IR (KBr) νmax/cm^−1^ 3221.35, 3387.83 (NH2); 1655.19 (CO); 1H NMR (500 MHz, DMSO-*d*_6_), 4: δ 5.842 (s, 2H, -NH2), 3.617 (s, 3H, phenyl-OCH3), 3.683 (s, 9H, phenyl-OCH3), 5.841 (s, 2H, -NH2), 7.013 (s, 2H, phenyl-H), 7.300–7.316 (d, 1H, *J* = 8.0 Hz, quinolone-*H*), 7.399–7.431 (m, 1H, quinazoline-H), 7.585–7.601 (d, 1H, *J* = 8.0 Hz, quinolone-*H*). 13C NMR (125 MHz, DMSO-*d*_6_) δ 55.62.56.06, 56.12, 60.12, 105.13, 115.21, 116.85, 121.78, 126.76, 127.36, 139.17, 140.00, 150.34, 152.79, 164.55, 162.26. MS (*m*/*z*): [M+1]: 358.1378.

8-methoxy-2-trimethoxyphenylquanazoline-4(3*H*)-one (**5**)

A mixture of 4*H*-1,3-Benzoxazin-4-one derivative (3, 3.43 g, 0.01 mol) and formamide (30 mL) was heated under reflux for 16 h. On cooling, the separated solid was filtered, washed with water, and recrystallized from EtOH; yield, 78%; mp: 242–244 °C; IR (KBr) νmax/cm^−1^ 1655.67 (CO); 1H NMR (500 MHz, DMSO-*d*_6_), 5: δ 2.488 (s, 3H, phenyl-CH3), 7.330 (d, 1H, *J* = 7.5 Hz, phenyl-H), 7.489 (d, 2H, *J* = 5.5 Hz, phenyl-H) 7.583 (d, 1H, *J* = 7.5 Hz, quinazoline-H), 7.758 (d, 1H, *J* = 7.5 Hz, quinazoline-H), 7.865 (d, 1H, *J* = 7.5 Hz, quinazoline-H), 8.083 (s, 1H, phenyl-H) 8.132 (s, H, phenyl-H) 8.292 (s, 1H, quinazoline-*N*H), 13C NMR (125 MHz, DMSO-*d*_6_) δ 16.53, 125.23, 125.43, 125.57, 128.03, 128.46, 128.68, 129.25, 130.83, 132.17, 133.65, 138.23, 152.28, 164.43, 170.12. MS (*m*/*z*): [M+1]: 343.1274.

3-methoxy-2-(trimethoxybenzamido) benzamide (**6**)

A mixture of 4*H*-1,3-benzoxazin-4-one derivative (3, 3.43 g, 0.01 mol) and ammonia solution (20 mL) was heated under reflux for 12 h. It was then poured onto water, filtered, dried, and recrystallized from dioxane; yield, 92%; mp: 132–134 °C; IR (KBr) νmax/cm^−1^ 3193.13, 3367.91(NH2), 3389.62 (NH), 1672.66, 1663.23 (CO); 1H NMR (500 MHz, DMSO-*d*_6_), 6: δ 3.760 (s, 3H, phenyl-OCH3), 3.860 (s, 9H, phenyl-OCH3), 6.069(s, 2H, -NH2), 6.478–6.509 (m, 1H, phenyl-H), 6.846 (d, 1H, *J* = 15.0 Hz, phenyl-H), 7.094 (d, 1H, *J* = 8.0 Hz, phenyl-H), 7.027–7.284 (m, 2H, phenyl-H) 9.782 (s, 1H, CO-NH). 13C NMR (125 MHz, DMSO-*d*_6_) δ 55.44, 55.85, 55.90, 59.99, 119.25, 122.66, 128.36, 138.47, 139.24, 139.59, 146.79, 147.40, 152.54, 165.40, 165.86, 168.41. MS (*m*/*z*): [M-1]: 361.1216.

3-methoxy-2-(trimethoxybenzamido)-benzoylhydrazide (**7**)

A mixture of 4H-1,3-benzoxazin-4-one derivative (3, 3.43 g, 0.01 mol) and hydrazine hydrate (85% 15 mL) in EtOH (30 mL) was heated under reflux for 2 h. The obtained solid was filtered, washed with water, dried, and recrystallized from EtOH; yield, 83%; mp: 195–197 °C; IR (KBr) νmax/cm^−1^ 3175.43, 3295.34,{NH2), 3376.45, 3382.21, (NH); 1670.63, 1655.19 (CO); 1H NMR (500 MHz, DMSO-*d*_6_), 7: δ 3.747 (s, 3H, phenyl-OCH3), 3.881 (s, 9H, phenyl-OCH3), 4.479 (s, 2H, -NH2), 7.051 (d, 1H, J 7.5 Hz, phenyl-H), 7.118 (d, 1H, *J* = 8.0 Hz, phenyl-H), 7.285–7.300 (d, 1H, *J* = 7.5 Hz, phenyl-H) 7.316–7.384 (m, 2H, phenyl-H) 10.134 (s, 1H, -CONHNH2), 10.167 (s, 1H, -NHCO). 13C NMR (125 MHz, DMSO*d*_6_) δ 56.03, 56.24, 60.11, 59.99, 105.33, 113.37, 120.42, 123.51, 127.89, 129.10, 134.73, 139.87, 152.45, 155.49, 164.79, 165.52. MS (*m*/*z*): [M−1]: 374.1349.

3-methoxy-2-(trimethoxybenzamido)-*N*-[benzenesulphonyl]benzoylhydrazide (**8**) 

A mixture of 3-methoxy-2-(trimethoxybenzamido)-benzoylhydrazide (7, 0.01 mol, 3.75 g) and benzene sulphonyl chloride (0.01 mol, 1.76 g) in 15 mL dioxane containing triethylamine (0.02 mol, 2.02 g) was refluxed for 5 h. The reaction mixture was cooled down and the solvent was removed under reduced pressure; the recovered solid was filtered, washed with water, dried, and recrystallized from ethanol; yield, 81%; mp: 263–265 °C; IR (KBr) νmax/cm^−1^ 3366.45, 3372.52, 3385.43 (NH), 1670.65, 1655.37 (CO); 1H NMR (500 MHz, DMSO-*d*_6_), 8: δ 3.629.(s, 3H, phenyl-OCH3), 3.942 (s, 9H, phenyl-OCH3), 7.281 (d, 1H, *J* = 5.0 Hz, phenyl-H),.7.319 (d, 2H, *J* = 8.0 Hz, phenyl-H), 7.359–7.374 (m, 3H, quinazoline-H), 7.390–7.441 (m, 2H, phenyl-H), 7.566 (2, 2H, *J* = 7.5 Hz, phenyl-H), 8.5497 (s, 1H, -CONHNH), 12.615 (s, 2H, -CONH). 13C NMR (125 MHz, DMSO-*d*_6_) δ 56.19.56.28, 60.18, 105.20, 119.38, 116.85, 121.78, 126.76, 127.36, 127.80, 139.12, 140.08, 150.42, 152.67, 164.36, 167.34. MS (*m*/*z*): [M−1]: 514.1275.

3-benzenesulfamoyl-8-methoxy-2-trimethoxyphenylquanazoline-4(3*H*)-one (**9**)

A mixture of 3-amino-8-methoxy-2-trimethoxyphenylquanazoline-4(3*H*)-one (4, 0.01 mol, 3.57 g) and benzene sulphonyl chloride (0.01 mol, 1.76 g) in 15 mL dioxane containing triethylamine (0.02 mol, 2.02 g) was refluxed for 6 h. The reaction mixture was cooled down and the solvent was removed under reduced pressure. The recovered solid was filtered, washed with water, dried, and recrystallized from ethanol; yield, 80%; mp: 244–246 °C; IR (KBr) νmax/cm^−1^ 3385.56 (NH), 1655.82 (CO); 1H NMR (500 MHz, DMSO-*d*_6_) 9: δ 187 3.632 (s, 3H, phenyl-OCH3), 3.748 (s, 9H, phenyl-OCH3), 6.7288–7.860 (m, 2H, phenyl-H), 7.224–7.290 (m, 3H, phenyl-H), 7.368–7.459 (m, 3H, phenyl-H), 7.480–7.537 (m, 3H, phenyl-H) 11.885 (s, 1H, NHSO2). 13C NMR (125 MHz, DMSO-*d*_6_) δ 56.15, 59.93, 107.04, 116.06, 117.31, 121.60, 126.11, 128.34, 132.61, 135.99, 138.28, 139.85, 151.63, 154.16, 154.43, 160.06. MS (*m*/*z*): [M−1]: 496.1171.

3-methyl-8-methoxy-2-trimethoxyphenylquanazoline-4(3*H*)-one (**10**) 

A mixture of 4*H*-1,3-benzoxazin-4-one derivative (3, 3.43 g, 0.01 mol) and methyl amine (0.096 g, 0.003 mol) in dry pyridine (15 mL) was heated under reflux for 18 h. The solvent was evaporated under reduced pressure. The obtained solid was filtered, washed with diluted HCl, and crystallized from glacial acetic acid; yield, 85%; mp: 218–220 °C; IR (KBr) νmax/cm^−1^ 1655.73 (CO); 1H NMR (500 MHz, DMSO-*d*_6_), 10: δ 2.637 (s, 3H,-CH3), 3.913 (s, 3H, phenyl-OCH3), 3.929 (s, 9H, phenyl-OCH3), 7.358 (d, 1H, *J* = 8.5 Hz, phenyl-H), 7.357–7.388 (m, 1H, quinazoline-H), 7.518 (s, 2H, phenyl-H), 7.641–7.656 (d, 1H, *J* = 7.5 Hz, quinazoline-H), 8.045 (d, 1H, *J* = 7.5 Hz, quinazoline-H). 13C NMR (125 MHz, DMSO-*d*_6_) δ 17.08, 56.31, 61.04, 59.99, 105.31, 116.59, 125.56, 126.19, 127.56, 135.97, 137.39, 141.87, 145.22, 153.23, 155.48, 160.16. MS (*m*/*z*): [M+1]: 357.1083.

8-methoxy-2-trimethoxymethyl-3-trimethoxyphenyl quanazoline-4(*3H*)-one (**11**) 

A mixture of 4*H*-1,3-benzoxazin-4-one derivative (3, 3.43 g, 0.01mol) and 3, 4, 5-trimethoxy aniline (1.89 g, 0.01 mol) in dry pyridine (20 mL) was heated under reflux for 16 h. The solvent was evaporated under reduced pressure. The obtained solid was filtered, washed with diluted HCl, and crystallized from glacial acetic acid; yield, 83%; mp: 237–239 °C; IR (KBr) νmax/cm^−1^ 1655.84 (CO); 1H NMR (500 MHz, DMSO-*d*_6_), 11: δ 3.624 (s, 3H, phenyl-OCH3), 3.768 (s, 9H, phenyl-OCH3), 3.964 (s, 9H, phenyl-OCH3), 7.103 (s, 2H, phenyl-H), 7.169 (s, 2H, phenyl-H) 7.248 (d, 1H, *J* = 8.5 Hz, quinazoline-H), 7.314 (d, 1H, *J* = 7.5 Hz, quinazoline-H), 7.429–7.445 (d, 1H, *J* = 8.0 Hz, quinazoline-H). 13C NMR (125 MHz, DMSO-*d*_6_) δ.56.18, 59.56, 60.23, 105.06, 119.63, 125.65, 126.05, 127.12, 129.32,129.65, 129.68, 129.97, 134.13, 134.34, 135.67, 145.96, 164.43, 165.34. MS (*m*/*z*): [M+1]: 509.1467.

### 3.2. Molecular Docking

The molecular docking was performed using Autodock Vina program [38]. The crystal structures of proteins (PDB code 6S9B for EGFR and 4ASD for VEGFR2) were prepared by eliminating unwanted co-crystalized ligands and water molecules using Discovery Studio Visualizer (Accelrys, USA). AutoDock Tools was employed to prepare the input pdbqt files for proteins and ligands and to set the size and the center of the grid box. The size of EGFR active site was set at 14 × 18 × 16 Å coordinates in x, y, and z dimensions and centered to (x = −51.787, y = 21.484, z= −0.026) using 1000 Å spacing. The size of VEGFR2 active site was set at 20 × 20 × 20 Å coordinates in x, y, and z dimensions and centered to (x = −23.756, y = −1.152, z = −11.701) using 1000 Å spacing. PyMol [37] and Discovery Studio Visualizer were used to analyze the binding mode and interaction of ligands with proteins.

### 3.3. Molecular Dynamic Simulation

All atom 20 ns molecular dynamics (MD) simulations was performed using GROMACS 2018.1 software [39]. OPLS-AA/L force field was used to generate the topology of proteins [40]. The topology and parameter of ligands were generated by the Swissparam server (available at http://www.swissparam.ch/, access on 17 May 2021) [41]. The MD simulations were performed using the previously reported method [42]. Briefly, the system was solvated in cubic box with TIP3P as a water model followed by adding counter ions to neutralize the system. Periodic boundary conditions were used during MD simulation. Energy minimization of system was performed using steepest descent algorithm with tolerance value of 1000 kJ mol^−1^ nm^−1^. The system was then equilibrated using NVT and NPT ensembles for 100 ps. Finally, 20 ns production MD was performed for the system, with trajectories generated every 2 femtoseconds (fs) and snapshots saved every 2 picoseconds (PS). Standard analysis was applied to calculate the root mean square deviation (RMSD) and hydrogen bond formation over the simulation time. Standard analysis was applied to calculate the root mean square deviation (RMSD) and hydrogen bond formation over the simulation time using gmx rms and gmx hbond, respectively.

### 3.4. Antitumor Screening

A primary anticancer assay was performed for two enzymes VEGFR2 and EGFR and 3 human tumor cell line panels, Hela, A549, and MDA, which are related to some neoplastic diseases, like cervical, lung, and breast carcinoma, in accordance with the protocol of the Drug Evaluation Branch, National Cancer Institute, Bethesda, MD [29,30,31,32,33].

## 4. Conclusions

A new series of 8-methoxy-2-trimethoxyphenyl-3-substituted quinazoline-4(*3H*)-one was synthesized and assessed for antitumor activity against three cell line panels, HeLa, A549, and MDA, using docetaxel as a reference drug. The most potent antitumor activity was found in compound 6, assessed as a VEGFR2 and EGFR enzyme inhibitor, with (IC50, 98.1 and 106 nM, respectively) relative to docetaxel (IC50, 89.3 and 56.1 nM, respectively) as reference drug. The molecular docking and 20 ns molecular dynamics (MD) simulation results suggest a stable binding of M1–6 within the ATP binding pocket of EGFR and VEGFR2 due to the stable hydrogen bonds formed with the proteins. That makes the results in line with the docking and in vitro results, supporting the notion that this compound might act as a dual EGFR and VEGFR2 tyrosine kinase inhibitor. Strong cytotoxic activity was shown for compounds 2, 6, 10, and 8 against Hela cell line with IC50 values of, 2.13, 2.8, 3.98, and 4.94 µM, respectively compared to docetaxel (IC50, 9.65 µM). Strong cytotoxic activity was shown for compound 11 against A549 cell line (IC50, 4.03 µM) in comparison to docetaxel (IC50, 10.8 µM). A strong cytotoxic activity was shown for compounds 6 and 9 against MDA cell line (IC50, 0.79 and 3.42 µM, respectively) relative to docetaxel (IC50, 3.98 µM).

## Figures and Tables

**Table 1:** IC50 [nM] values for the cytotoxic effect of compound 6 and docetaxel on enzymes VEGFR2 and EGFR.

Compound			Results	
			VEGFR2	*EGFR*
Compound No.	Code	MW	IC50nM	IC50nM
6	M6	360	98.1 ± 2.93	106 ± 2.22
Reference	Docetaxel	807.88	89.3 ± 2.67	56.1 ± 1.17

**Table 2:** IC50 [µM] values of compounds **2**–**11** and docetaxel.

Compound No.	Group M		Cytotoxicity IC50 µM		
	Code	MW	Hela	A549	MDA
4	M-1	357	29.8 ± 0.78	18 ± 0.5	4.12 ± 0.1
3	M-2	343	66.5 ± 1.74	43.6 ± 1.1	17.4 ± 0.5
11	M-3	506	12.0 ± 0.31	4.03 ± 0.1	7.06 ± 0.2
2	M-4	361	2.13 ± 0.06	29.7 ± 0.8	9.24 ± 0.2
5	M-5	342	12.9 ± 0.34	26.3 ± 0.7	4.39 ± 0.1
6	M-6	360	2.8 ± 0.07	81 ± 2.1	0.79 ± 0.04
7	M-7	375	18.7 ± 0.49	46.3 ± 1.2	9.0 ± 0.2
8	M-8	515	4.94 ± 0.13	13 ± 0.3	11.7 ± 0.3
9	M-9	497	11.7 ± 0.31	19.9 ± 0.5	3.42 ± 0.1
10	M-10	356	3.98 ± 0.1	12.3 ± 0.3	7.77 ± 0.2
Ref.	Docetaxel	807.8	9.65 ± 0.2	10.8 ± 0.23	3.98 ± 0.08

## Data Availability

Not applicable.

## Sample Availability

Samples of the compounds are not available from the authors.
